# Author Correction: The Ca^2+^-activated chloride channel anoctamin-2 mediates spike-frequency adaptation and regulates sensory transmission in thalamocortical neurons

**DOI:** 10.1038/s41467-021-26866-9

**Published:** 2021-11-05

**Authors:** Go Eun Ha, Jaekwang Lee, Hankyul Kwak, Kiyeong Song, Jea Kwon, Soon-Young Jung, Joohyeon Hong, Gyeong-Eon Chang, Eun Mi Hwang, Hee-Sup Shin, C. Justin Lee, Eunji Cheong

**Affiliations:** 1 Department of Biotechnology, College of Life Science and Biotechnology, Yonsei University, Seoul, 03722 Republic of Korea; 2 Center for Neural Science, Korea Institute of Science and Technology, Seoul, 02792 Republic of Korea; 3 Center for Functional Connectomics, Korea Institute of Science and Technology, Seoul, 02792 Republic of Korea; 4 Center for Cognition and Sociality, Institute for Basic Science, Daejeon, 34141 Republic of Korea; 5 KU-KIST Graduate School of Converging Science and Technology, Korea University, 145 Anam-ro, Seongbuk-gu, Seoul, 34141 Republic of Korea

Correction to: *Nature Communications* 10.1038/ncomms13791, published online 19 December 2016.

The original version of this Article omitted one affiliation for two of the authors, Jea Kwon and C. Justin Lee. The omitted affiliation is ‘^5^KU-KIST Graduate School of Converging Science and Technology, Korea University, 145 Anam-ro, Seongbuk-gu, Seoul, 02841 Republic of Korea’. This has been corrected in both the PDF and HTML versions of the Article.

