# Supplementary material for: The Ca2+-activated chloride channel anoctamin-2 mediates spike-frequency adaptation and regulates sensory transmission in thalamocortical neurons
Source: Nat Commun. 2016 Dec 19;7:13791. doi: 10.1038/ncomms13791 (PMC5187435; doi:10.1038/ncomms13791)
Supplement: Supplementary Information — Supplementary Figures, Supplementary Table, Supplementary Methods, Supplementary Notes and Supplementary References. [file ncomms13791-s1.pdf]

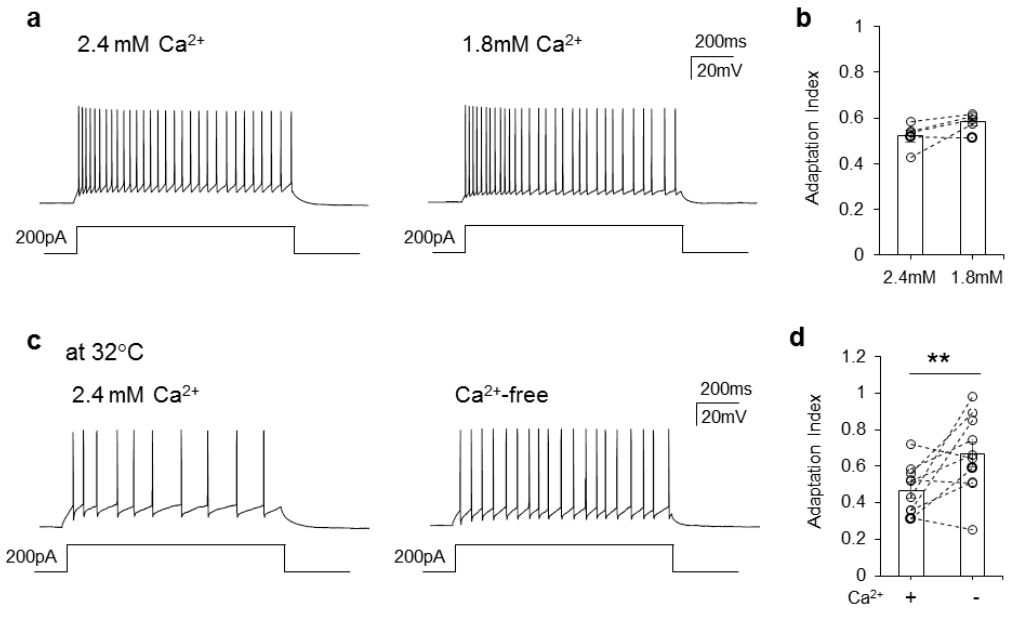

Supplementary Figure 1. Spike-frequency adaptation in TC neurons under physiological conditions (**a**) A depolarizing current of 100 pA induces tonic firing in TC neurons in 2.4 mM Ca^{2+} buffer followed by replacement with 1.8 mM Ca^{2+} buffer, a more physiological concentration. (**b**) The adaptation index was not different between 2.4 mM Ca^{2+} and 1.8 mM Ca^{2+} buffer. (**c**) A depolarizing current of 100 pA induces tonic firing in TC neurons in 2.4 mM Ca^{2+} buffer followed by replacement with Ca^{2+} -free buffer at 32°C. (**d**) The adaptation index was significantly increased (*p<0.05, paired *t*-test, n=10 from 4 mice).

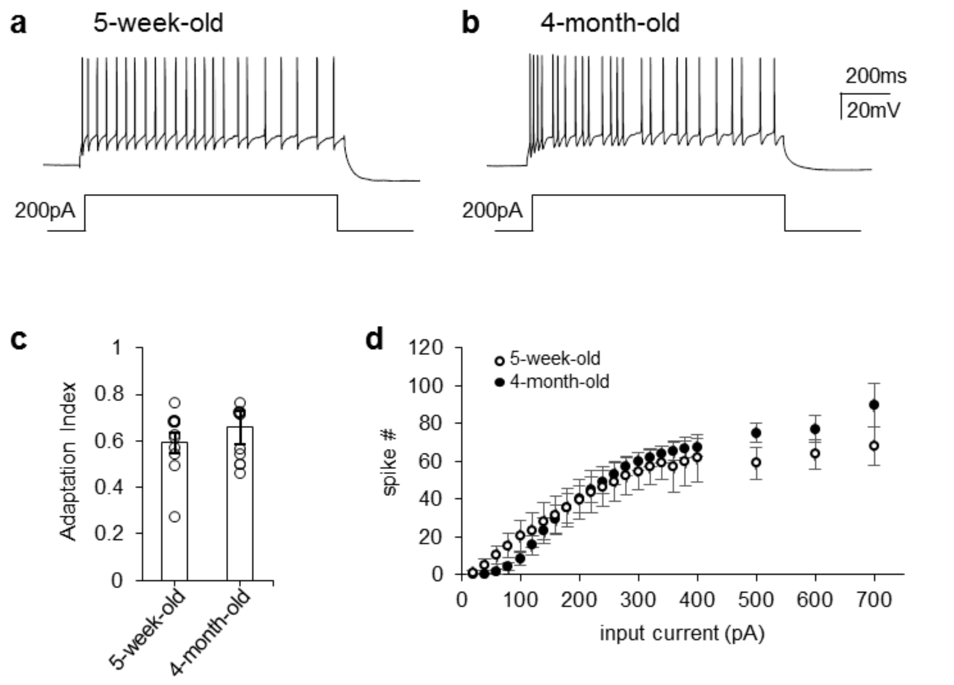

Supplementary Figure 2. Spike-frequency adaptation and firing rates of TC neurons from 5-week- and 4-month-old mice (**a-b**) Tonic firing in TC neurons from 5-week- and 4-month-old mice was induced by 200 pA depolarizing current. (**c**) The adaptation index between TC neurons of 5-week-old (n=10 from 5 mice) and 4-month-old mice (n=9 from 3 mice) was not different. (**d**) Input-output curves show that firing rates under various current injections of TC neurons from 5-week-old mice were similar to those from 4-month-old mice.

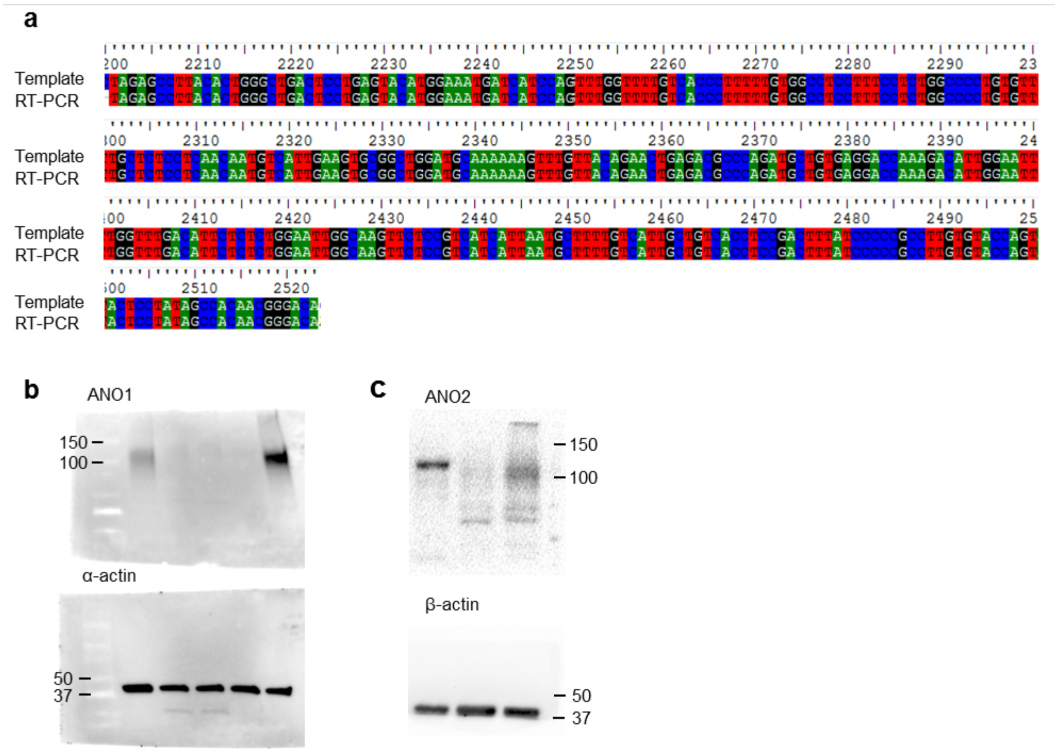

Supplementary Figure 3. Sequencing data for the *ANO2* RT-PCR product and full scans of western blots (**a**) Alignment image showing complete correspondence between sequencing data from the *ANO2* RT-PCR product and the template (**b-c**) Full blotting images of Figure 5j (b) and Figure 6b (c)

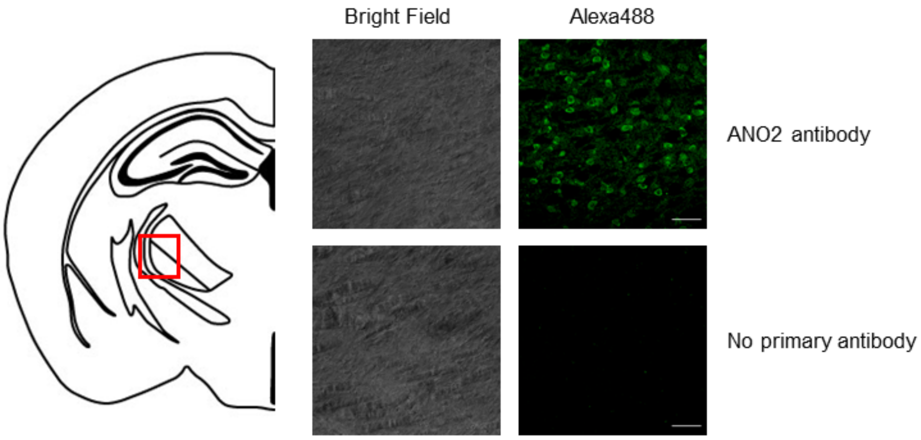

Supplementary Figure 4. Specificity of secondary antibody for detection of anti-ANO2 antibody Immunohistochemical image in VB region using Alexa488 goat anti-rabbit secondary and rabbit anti-ANO2 primary antibodies. The Alexa488 signal was detectable in conditions where both antibodies were included, but not in conditions where only the secondary antibody was used. Therefore, the Alexa488 goat anti-rabbit antibody is specific for detecting rabbit antibodies.

a

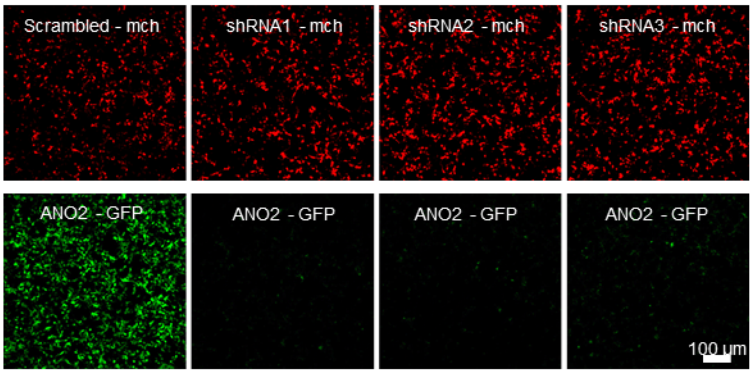

b

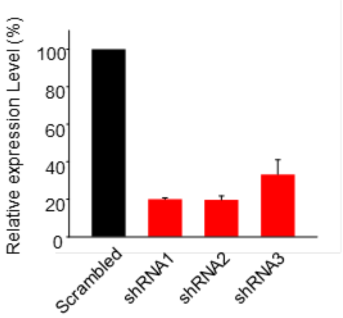

Expression Level (%)

Scr	shRNA1	shRNA2	shRNA3
100	20.0	19.7	33.3

Supplementary Figure 5. Development and validation of *ANO2* shRNA (**a**) Confocal images of HEK293T cells transfected with three different shRNA candidates, as well as scrambled shRNA, indicating that the ANO2 full clone expressed in HEK293T cells was knocked down by shRNA transfection. (**b**) Bar graph displaying relative expression of *ANO2*.

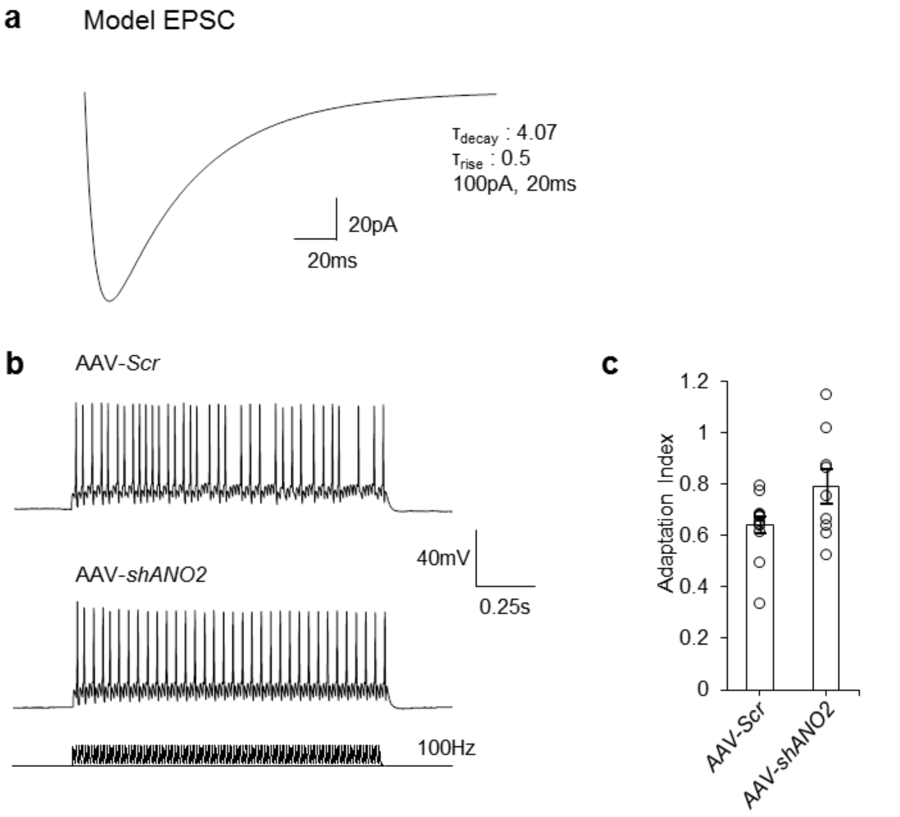

Supplementary Figure 6. Spike-frequency adaptation in TC neurons stimulated with EPSC-like stimulation (**a**) A model EPSC was generated from a double exponential equation of EPSCs, as detailed in Supplementary Methods. Model EPSCs had widths of 20 ms and various amplitudes ranging from 200 pA to 1000 pA. (**b**) EPSC-like stimulation at 100 Hz induced a firing frequency adaptation pattern in AAV-*Scr*-infected TC neurons, while TC neurons infected with AAV-*shANO2* exhibited an attenuated adaptation pattern. (**c**) The adaptation index indicated that spike frequency adaptation of TC neurons was decreased by knockdown of ANO2, although not significantly (AAV-*Scr* n=13 from 5 mice, AAV-*shANO2* n=9 from 5 mice).

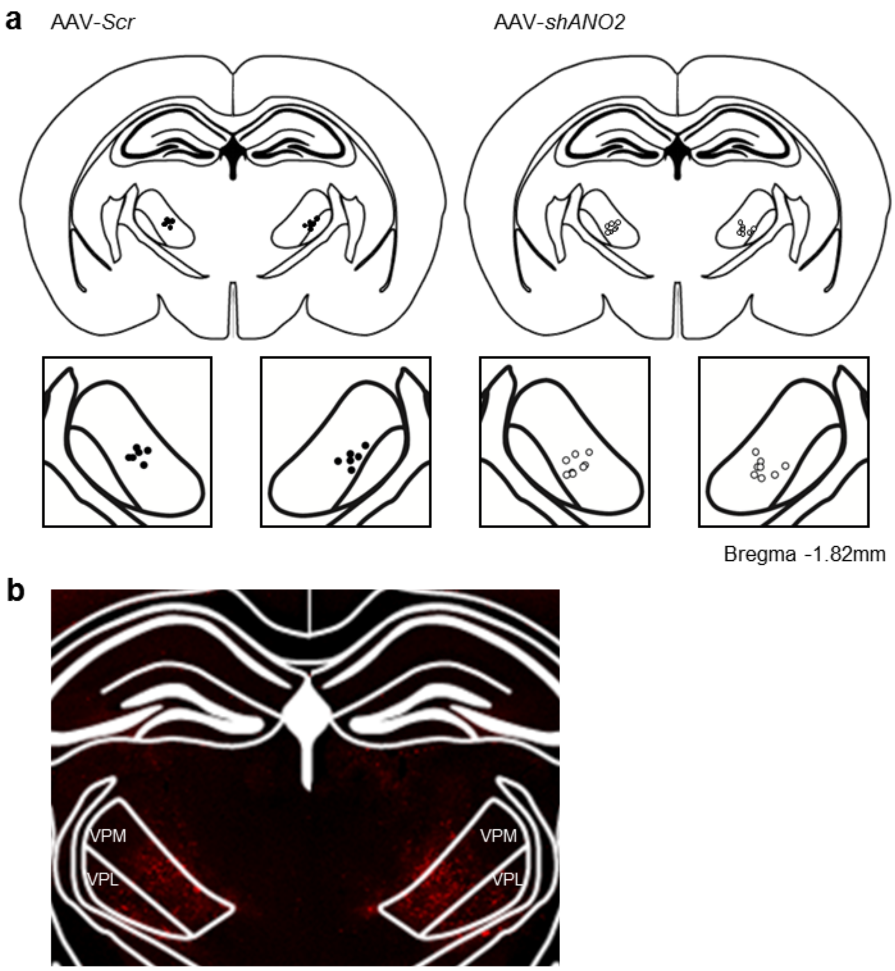

Supplementary Figure 7. Injection site and spread of AAVs (**a**) The viral injection sites in the VB region of the thalamus are shown in the whole mouse brain atlas (upper) and the enlarged view of the thalamus (bottom) at Bregma -1.82 mm. Both AAV-*Scr* and AAV-*shANO2* were accurately targeted to the VB region. (**b**) mCherry expression, which reported viral expression, confirmed the extent of the AAV-infected area in individual mice. Mice with at least 57.8% of the VB region infected (70.2 ± 3.9%, n=32) were included for further behavioral analyses.

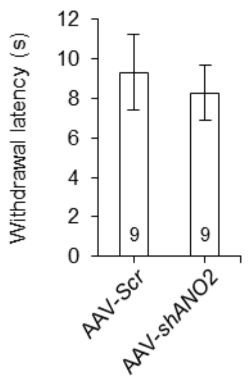

Supplementary Figure 8. Acute pain responses measured on the hot plate test

Differences in withdrawal latency on the hot plate test were not observed between AAV-*Scr*-injected (n=9) and AAV-*shANO2*-injected mice (n=9).

Supplementary Table 1. Membrane properties of TC neurons and CA1 neurons recorded

	RMP (mV)	C_m (pF)	R_{in} (MΩ)
2.4mM [Ca^{2+}]	-67.1±1.3	147.3±13.9	214.1±37.0
1.8mM [Ca^{2+}]	-68.0±1.41	155.2±15.6	264.3±60.8
AAV-*Scr*	-66.7±1.15	120.58±6.71	238.66±32.93
AAV-*shANO2*	-66.9±1.06	123.27±5.39	257.33±19.7
13-week-old	-64.4±1.3	165.2±13.4	235.5±31.6
CA1 AAV-*Scr*	-67.7±0.86	141.7±24.2	160.3±15.34
CA1 AAV-*shANO2*	-66.1±1.13	151.19±19.8	160.39±18.93

Supplementary Methods

Excitatory postsynaptic current (EPSC)-like stimulation

To generate a model of EPSCs, we used a double exponential equation, as shown in equations (1) and (2). Rise (τ_{rise}) and decay (τ_{decay}) tau values were adopted from references that measured the kinetics of EPSCs in VB neurons with patch clamp recordings[1]. τ_{rise} and τ_{decay} values were 0.5 and 4.07, respectively. A model EPSC trace is shown in Supplementary Fig. 6a. A single model EPSC had a 20 ms width with various amplitudes ranging from 200 to 1000 pA, and trains of 100 Hz EPSC-like stimuli were delivered to VB neurons infected by either AAV-*Scr* or AAV-*shAno2* viruses.

Equation (1) $A(t) = A_1 e^{(-\frac{t}{\tau_{rise}})} - A_2 e^{(-\frac{t}{\tau_{decay}})}$

Equation (2) $t_{peak} = t_0 + \frac{\tau_{decay}\tau_{rise}}{\tau_{decay}-\tau_{rise}} \ln(\frac{\tau_{decay}}{\tau_{rise}})$

Responses to EPSC-like stimuli were measured under current clamp in aCSF solution (NaCl, 124 mM; KCl, 3 mM; $MgSO_4$, 6.5 mM; NaH_2PO_4, 1.25 mM; $NaHCO_3$, 26 mM; $CaCl_2{\cdot}2H_2O$, 1 mM; and glucose, 10 mM) aerated with 95% O_2/5% CO_2. Patch electrodes (4-6 MΩ) fabricated from standard-wall borosilicate glass (GC150F-10, Warner Instrument Corp., USA) were filled with an intrapipette solution containing K-gluconate, 125 mM; KCl, 10 mM; $MgCl_2$, 1 mM; HEPES, 10 mM; EGTA, 0.02 mM; Mg-ATP, 4 mM; and Na_2-GTP, 0.3 mM, with pH adjusted to 7.35.

shRNA design and vector construction

The mouse anoctamin-2 (m*ANO2*) nucleotides (NM_153589) from 570-590 (5'-GCATGCACTTTCACGACAACC-3'), from 1137-1157 (5'-GCCGCATTGTACACG-AGATTC-3'), and from 1750-1770 (5'-GGAACACTCTCGGCCTGAATA-3') were selected as the shRNA target regions. The pENSR-shANO2-mCherry construct was

synthesized using the following complementary oligomers: shRNA1, 5'-agagaGGTTGTCGTGAAAGTGCATGCtttttctcgagtactagga-3' (sense), 5'-tgaaGG-TTGTCGTGAAAGTGCATGCaaacaaggcttttctccaag-3' (antisense); shRNA2, 5'-agagaGAATCTCGTGTACAATGCGGCtttttctcgagtactagga-3'(sense), 5'-tgaaGAATC-TCGTGTACAATGCGGCaaacaaggcttttctccaag-3' (antisense); shRNA3, 5'-agaga-TATTCAGGCCGAGAGTGTTCCtttttctcgagtactagga-3' (sense), 5'-tgaaTATTCAGGC-CGAGAGTGTTCCaaacaaggcttttctccaag-3' (antisense). The oligomers were inserted into pENSR backbones by site-directed mutagenesis (Enzynomics, Korea) and verified by sequencing. A scrambled shRNA-containing construct was used as a control: 5'-ttcgcatagcgtatgccgttttcaagagaaac-ggcatacgctatgcgatttttc-3' (sense), 5'-tcgagaaaaaatcgcatagcgtatgccgtttctcttgaaaacg-gcatacgctatgcgaa-3' (antisense).

Quantitative RT-PCR (qRT-PCR) analysis of ANO2 knockdown in HEK293T cells

HEK293T cells were transfected with the pENSR vector carrying *ANO2* shRNA to silence ANO2. Approximately 3 d after transfection, total RNA was prepared using TRIzol Reagent (Invitrogen, USA), and cDNA was synthesized using Super Script III Reverse Transcriptase (Invitrogen, USA). The qRT-PCR primers used to assess expression of mANO2 in cDNA from ANO2 transfected HEK293T cells were as follows: mANO2 forward, 5'-aggctgtctcatggagctgt-3' and reverse, 5'-tgctctggacgttttgagtg-3'.

Hot plate test

The hot plate test, which measures acute pain sensitivity, was conducted using an electronically controlled hot plate meter heated to 53.5°C. Mice were placed on the hot plate enclosed by a circular plastic container. The latency to respond by licking paws or jumping was measured, and the maximum duration of the test was 30 s.

Supplementary Note 1. Spike-frequency adaptation in VB neurons under physiological condition

Extracellular Ca^{2+} concentration could influence neuronal activity by modulating Ca^{2+} influx through voltage-gated Ca^{2+} channels and the properties of synaptic receptors, such as nicotinic acetylcholine[2] and glutamatergic receptors. We observed spike-frequency adaptation mediated by Ca^{2+} influx in 2.4 mM $[Ca^{2+}]_{ex}$ (Fig. 1), which is relatively high. To examine whether spike-frequency adaptation also occurs under more physiological $[Ca^{2+}]_{ex}$, we measured the firing pattern of TC neurons in extracellular buffer containing 1.8 mM Ca^{2+}, which is within the physiological range of the mammalian brain (1.5 mM to 2.0 mM)[3]. Replacement of the 2.4 mM Ca^{2+} extracellular buffer with 1.8 mM Ca^{2+} did not change the firing pattern of TC neurons (Supplementary Fig. 1a-b). The adaptation index was also unaltered by the replacement of 2.4 mM with 1.8 mM Ca^{2+} buffer (0.52 ± 0.03 vs. 0.58 ± 0.02, Supplementary Fig. 1c). Therefore, we decided to use extracellular buffer containing 2.4 mM Ca^{2+} to increase the effects on Ca^{2+}-activated channels and induce spike-frequency adaptation, as seen under physiological conditions.

Temperature could influence various cellular processes. Thus, we investigated whether spike-frequency adaptation is affected by temperature. We obtained tonic firing of TC neurons at 32℃ recording temperature in extracellular buffer containing 2.4 mM Ca^{2+}, followed by Ca^{2+}-free buffer (Supplementary Fig. 1c-d). The adaptation index was similarly altered by the replacement of 2.4 mM Ca^{2+} buffer with Ca^{2+}-free buffer (0.47 ± 0.04 vs. 0.66 ± 0.07) as the values obtained at 25℃ (Fig. 1).

Supplementary Note 2. Comparison of spike-frequency adaptation in VB neurons of 5-week-old and 4-month-old mice

It was previously reported that thalamocortical (TC) neurons in the lateral dorsal thalamic nucleus of 15-day-old mice have attenuated firing frequencies in response to depolarizing stimuli compared to those of 3–7-month-old mice[4]. To investigate whether 5-week-old mice that were used in this study also had different firing properties when compared with older mice, we measured spike-frequency adaptation of TC neurons in 5-week-old and 4-month-old mice (Supplementary Fig. 2a-b). Analysis of the adaptation index revealed that there was no difference between 5-week-old and 4-month-old mice (0.59 ± 0.04, n=10 from 5 mice vs. 0.66 ± 0.07, n=9 from 3 mice, with 200 pA stimulation, Supplementary Fig. 2c). The input-output curve showed that there were no significant differences in firing rates of TC neurons from 5-week-old and 4-month-old mice with depolarizing current steps up to 400 pA (Supplementary Fig. 2d). Stronger depolarizing current steps ranging from 500 to 700 pA induced further increases in firing frequencies of TC neurons from 4-month-old mice, whereas the firing frequencies of TC neurons in 5-week old mice were not substantially increased.

Supplementary Note 3. The ANO2 RT-PCR product corresponded to the ANO2 sequence

Sequencing data of the *ANO2* RT-PCR product obtained from thalamic mRNA completely aligned with the *ANO2* sequence (NCBI Reference Sequence: NM_153589.2; Supplementary Fig. 3a), confirming that *ANO2* was expressed in the thalamus.

Supplementary Note 4. Specificity of secondary antibody

Alexa488 goat anti-rabbit IgG, which was used as a secondary antibody for the rabbit anti-ANO2 primary antibody, was tested to confirm specificity. Alexa488 signal was not detected after immunostaining without the anti-ANO2 antibody; however, Alexa488 signal was detected after immunostaining with both anti-ANO2 and anti-rabbit IgG antibodies (Supplementary Fig. 4a); this indicates that the secondary antibody used for detection of the ANO2 antibody is specific for rabbit IgG.

Supplementary Note 5. ANO2 shRNA knocked down mANO2 expression in HEK293T cells

The efficacy of mANO2 shRNAs was tested by transfecting ANO2 shRNA into HEK293T cells expressing ANO2 tagged with IRES-GFP (Supplementary Fig. 5a). We selected three shRNA sequences targeting *Ano2* and cloned them into our vector. Silencing of the *ANO2* gene was quantified by quantitative RT-PCR (qRT-PCR) (Supplementary Fig. 5b). Of the three shRNAs, shRNA2 had the greatest efficacy, and was therefore inserted into an AAV transfer vector that contained the red fluorescent protein *mCherry* gene, enabling use in subsequent experiments

Supplementary Note 6. EPSC-like stimuli induces spike-frequency adaptation in VB neurons modulated by ANO2 channels

We examined the role of ANO2 in spike-frequency adaptation induced by EPSC-like stimuli, as previously reported[4]. First, we developed a model of EPSCs that mimicked EPSCs of VB neurons by using a double exponential equation, as detailed in Supplementary Methods (Supplementary Fig. 6a). The firing response to the continuous EPSC-like currents at 100 Hz could induce spike frequency adaptation in

TC neurons infected by AAV-*Scr* (Supplementary Fig. 6b, top panel). However, AAV-*shANO2*-infected VB neurons showed an increased adaptation index compared to AAV-*Scr*-infected VB neurons (0.64 ± 0.03, n=13 from 5 mice vs. 0.78 ± 0.07, n=9 from 5 mice, Supplementary Fig. 6b-c), which suggested that spike frequency adaptation under physiological conditions was also generated by the activation of ANO2 channels.

Supplementary Note 7. Determination of viral brain injection sites after visceral pain test

After behavioral testing, the brains from mice injected with either AAV-*Scr* or AAV-*shANO2* were isolated and cryo-sectioned to confirm both the viral injection site and the infectivity. AAV-*Scr* and AAV-*shANO2* were accurately injected into VB regions of the thalamus (Supplementary Fig. 7a), and the spread of the virus was determined by analyzing the ratio of the mCherry-expressing area to the total VB regional area (Supplementary Fig. 7b). Data from behavioral analyses of the visceral pain and hot plate tests were obtained from mice that had, on an average, a 70.2 ± 3.9% infection ratio in the VB region.

Supplementary Note 8. Knockdown of ANO2 in VB neurons did not alter acute pain responses

To investigate whether knockdown of ANO2 in TC neurons in VB nuclei influenced acute pain responses, we used the hot plate test to estimate thermal nociception of AAV-injected mice. Acute pain responses were analyzed by measuring the latency to lick paws or jump up, known as the “withdrawal latency”. There was no difference in withdrawal latency between AAV-*Scr*-injected and AAV-*shANO2*-injected mice

(Supplementary Fig. 8), which indicates that spike frequency adaptation mediated by ANO2 in TC neurons does not significantly contribute to controlling acute pain.

References

1. Miyata, M. & Imoto, K. Different composition of glutamate receptors in corticothalamic and lemniscal synaptic responses and their roles in the firing responses of ventrobasal thalamic neurons in juvenile mice. *J. Physiol.* **575**, 161-174 (2006).

2. Lena, C. & Changeux, J. P. Role of Ca2+ ions in nicotinic facilitation of GABA release in mouse thalamus. *J. Neurosci.* **17**, 576-585 (1997).

3. Egelman, D. M. & Montague, P. R. Calcium dynamics in the extracellular space of mammalian neural tissue. *Biophys. J.* **76**, 1856-1867 (1999).

4. Kasten, M. R., Rudy, B. & Anderson, M. P. Differential regulation of action potential firing in adult murine thalamocortical neurons by Kv3.2, Kv1, and SK potassium and N-type calcium channels. *J. Physiol.* **584**, 565-582 (2007).
